# New Appliances and Things Medical

**Published:** 1903-10-31

**Authors:** 


### NEW APPLIANCES AND THINGS MEDICAL.

[We shall be glad to receive at our Office, 28 & 29 Southampton Street, Strand, London, W.C., from the manufacturers, specimens of all new preparations and appliances which may be brought out from time to time.]

### ROSBACH WATER.

The address of this water was wrongly given in our notice last week. It should have been Rosbach, 81 Commercial Road, Lambeth, S.E.

### COOK'S SOAPS.

(E. Cook & Co., Ltd., Bow, London, E.)

We have previously had the opportunity of examining many of Cook's toilet and medicated soaps, and we have formed the same high opinion of their latest specialities. Cambath Soap, Dorina Soap, Coal Tar Soap, and Carbolic Soap are all excellent pure soaps, giving creamy lathers devoid of free alkali and irritating qualities. Special mention must be made of "Lasso" soap which contains a grease-removing constituent, and is therefore adapted for the use of cyclists, motorists, engineers, etc., who require something more than the detergent effect of ordinary soap. We find that it answers well to its claims and should be a most useful requisite in the household.

### FRY'S COCOA AND MILK.

(J. S. Fry and Sons, Limited, Bristol).

An examination of the chemical composition of cocoa might lead one to suppose that it was of considerable nutritive value, and the public usually regard it as a valuable food. Theoretically it is so, but practically it is not, as so little of it can be taken at a time. However, when the beverage is prepared entirely with milk and plenty of sugar, cocoa becomes an important food, but that is due to the milk and sugar and not to the cocoa. The preparation before us is an excellent combination of Fry's pure cocoa (which contains no added starch) with the finest sweetened milk, which with boiling water makes a most palatable, nourishing drink. The convenience of this preparation should prove very acceptable to many.

